# Plant responses to elevated temperatures: a field study on phenological sensitivity and fitness responses to simulated climate warming

**DOI:** 10.1111/gcb.12430

**Published:** 2013-11-19

**Authors:** David A Springate, Paula X Kover

**Affiliations:** * School of Life Sciences, University of ManchesterManchester, M13 9PL, UK; † Department of Biology and Biochemistry, University of BathBath, BA2 7AY, UK

**Keywords:** *Arabidopsis thaliana*, flowering time, natural variation, phenological sensitivity, plasticity

## Abstract

Significant changes in plant phenology have been observed in response to increases in mean global temperatures. There are concerns that accelerated phenologies can negatively impact plant populations. However, the fitness consequence of changes in phenology in response to elevated temperature is not well understood, particularly under field conditions. We address this issue by exposing a set of recombinant inbred lines of *Arabidopsis thaliana* to a simulated global warming treatment in the field. We find that plants exposed to elevated temperatures flower earlier, as predicted by photothermal models. However, contrary to life-history trade-off expectations, they also flower at a larger vegetative size, suggesting that warming probably causes acceleration in vegetative development. Although warming increases mean fitness (fruit production) by ca. 25%, there is a significant genotype-by-environment interaction. Changes in fitness rank indicate that imminent climate change can cause populations to be maladapted in their new environment, if adaptive evolution is limited. Thus, changes in the genetic composition of populations are likely, depending on the species’ generation time and the speed of temperature change. Interestingly, genotypes that show stronger phenological responses have higher fitness under elevated temperatures, suggesting that phenological sensitivity might be a good indicator of success under elevated temperature at the genotypic level as well as at the species level.

## Introduction

Flowering time can affect many aspects of a plant's ecology and fitness (Rathcke & Lacey, 1985; Parra-Tabla & Vargas, 2004; Kover *et al.*, 2009a; Amasino, 2010). Accordingly, natural variation in flowering time has been shown to be under selection (Le Corre *et al.*, 2002; Franks *et al.*, 2007; Korves *et al.*, 2007; Anderson *et al.*, 2011; Munguía-Rosas *et al.*, 2011). Mean global temperatures have risen by around 0.8 °C in the last hundred years and further increases of 2–3 °C are expected by the end of the century (Minorsky, 2002; IPCC, 2007). Climate change is expected to have its strongest and most immediate effects on plant phenology (Forrest & Miller-Rushing, 2010; Munguía-Rosas *et al.*, 2011), and accelerated phenologies have already been observed in many species (Sparks *et al.*, 2000; Abu-Asab *et al.*, 2001; Menzel *et al.*, 2006; Cleland *et al.*, 2012). There is concern that accelerated phenologies may alter patterns of resource allocation, interactions with pollinators, the size and diversity of the soil seed bank and compromise species persistence (Visser & Holleman, 2001; Minorsky, 2002; Walther *et al.*, 2002; Post & Pedersen, 2008; Hegland *et al.*, 2009). Here, we use a climate manipulation experiment under field conditions, to investigate the consequences of elevated temperature to flowering time and fitness in the plant *Arabidopsis thaliana*.

The use of environmental cues to flower at the right time is critical to capitalize on the best environmental conditions to produce fruits. It has been suggested that species that do change their phenology in response to climate change (phenologically sensitive species) are better at tracking optimal environmental conditions, and therefore more likely to persist (Cleland *et al.*, 2012). Accordingly, phenological sensitivity has been recently incorporated in species vulnerability assessments (Glick *et al.*, 2011). Although phenological sensitivity at the population or species level must be connected to responses at the individual level, a connection between this proposed species-level phenomenon and a mechanistic understanding at the population and genotypic level has not been investigated (Forrest & Miller-Rushing, 2010). ‘Phenological sensitivity’ is a concept analogous to ‘flowering plasticity’, which is the difference in flowering time expressed by the same genotype under different environmental conditions. Although phenotypic plasticity is very common in plants, its adaptive value and its role in facilitating plants coping with environmental change is much debated (Ghalambor *et al.*, 2007; Valladares *et al.*, 2007; Nicotra *et al.*, 2010). Here, we investigate the genetic architecture of plastic responses, and test specifically whether genotypes that are more phenologically responsive to temperature (show higher plasticity in flowering time) are better adapted to changes in climate.

Life-history theory predicts that early flowering genotypes (i.e. genotypes that flower earlier in relation to planting date) will transition into reproduction at smaller vegetative size, reducing their reproductive output (Mitchell-Olds, 1996). Therefore, if earlier flowering in response to climate warming is achieved by earlier onset of reproductive development; we would also expect a reduction in both vegetative size and fitness. However, if in response to climate change, plants transition to flowering earlier due to a general increase in the rate of vegetative development (because climate change improves the quality of the environment), plants can both flower earlier and be larger (therefore increasing their fitness). A better understanding of the mechanism through which flowering is accelerated in response to elevated temperature would clarify whether there is a need for concern. Yet, studies that combine responses to temperature and life-history trade-offs are rare (Metcalf & Mitchell-Olds, 2009).

Flowering time is a complex trait that is affected by a range of environmental factors such as photoperiod, ambient temperature, vernalization and plant size (Boss *et al.*, 2004; Cockram *et al.*, 2007; Colasanti & Coneva, 2009). Many of these studies were carried out in the model plant *Arabidopsis thaliana* because this species is easily manipulated experimentally, amenable to the construction of inbred lines and genetically well characterized (e.g. Johanson *et al.*, 2000; Lempe *et al.*, 2005; Wilczek *et al.*, 2010). More than 60 genes have been identified to affect flowering time in *A. thaliana*, including genes that affect the thermosensory pathway (Blazquez *et al.*, 2003; Balasubramanian, 2006) under laboratory conditions. However, little is known about the importance of small changes in average temperature under field conditions, or the genetic basis of responses to such changes. The few studies on *A. thaliana* performed under field conditions (e.g. Weinig *et al.*, 2002; Brachi *et al.*, 2010) suggest that plants in the field can respond very differently from laboratory experiments because they simultaneously experience a larger number and a wider range of environmental cues (including variations in light quality, temperature and photoperiod). Because under field conditions, plants in different locations will experience different photoperiods and temperatures, photothermal models that incorporate both photoperiod and temperature data have been proposed to translate flowering time measured in days into photothermal units (taking into account local environmental conditions). It is hypothesized that plants integrate photoperiod and thermal cues to transition into reproduction once a genetically determined threshold of accumulated photothermal units has been reached. These models have been used successfully to study the importance of different mutants and genetic pathways in *A. thaliana*(Wilczek *et al.*, 2009), and they suggest that for a given site (where the photoperiod is a constant), flowering time should be a linear function of temperature.

Field studies on the effect of climate warming on phenology typically compare populations or species over long periods of time or across environments (e.g. Fitter & Fitter, 2002; Wilczek *et al.*, 2010; Ågren & Schemske, 2012). However, because flowering is affected by both temperature and photoperiod, among other variables, comparisons across sites and time periods cannot clearly separate the effect of temperature. Here, we used surface-level heating cables (as championed by Grime *et al.*, 2000, 2008) to investigate the effect of temperature on a set of *A. thaliana* recombinant inbred lines. Such climate manipulation under field conditions is particularly powerful because it allows plants to be exposed to small elevations in temperatures without losing information about daily variation in temperature, day length, or other environmental cues, which will be equal across treatments. The use of *A. thaliana* mapping lines allows us to also explore the underlying genetic basis and the genetic variation in response to elevated temperature. Using this approach, we ask the following questions:

1. Are small changes in temperature sufficient to change the phenology and fitness of *A. thaliana* given all other environmental cues in the field?
2. What is the genetic architecture of the plastic response in flowering, vegetative size and fitness to elevated temperature?
3. Does acceleration in phenology in response to elevated temperatures cause smaller vegetative size and compromise fitness?
4. Do more phenologically responsive genotypes have higher fitness under elevated temperatures?

## Materials and methods

### Experimental design

We used a set of 320 Multiparental Advanced Genetic InterCross (MAGIC) *A. thaliana* lines (Kover *et al.*, 2009b). These nearly isogenic lines are derived from an outbred population composed of 19 natural accessions of *A. thaliana* that have been genotyped with 1260 single nucleotide polymorphisms (SNPs). Thus, they can be used for QTL mapping as well as to estimate response to the environment. That is because replicates of each MAGIC line are nearly genetically identical, allowing an estimation of the average difference in phenotype under different environments, i.e. their ‘plasticity’. For each MAGIC line, we prepared 10 microtubes, each containing five seeds in a 0.2% agar solution. All tubes were cold stratified for 7 days to promote synchronized germination. All seeds from each tube were directly planted into the soil in April 2009 with the help of a pipette into one of 10 plots set-up at the Botanical research station of the University of Manchester (UK). Multiple seeds were used to ensure we have at least one successful germinant per planting.

Each plot was 320 by 100 cm, and contained a single replicate of each line. Seeds and agar mixture were planted into a 5 mm diameter plastic ring pressed into the surface of the soil at 10 cm intervals to mark planting positions. The position of each line within a plot was randomly assigned. An 8 by 12 m fruit cage was erected over the 10 plots to protect the experiment from herbivores. Plots were arranged in two rows of five plots, and every other plot was assigned to a warming treatment (forming a reticulate pattern). Warming cables (600W; Thermoforce HQ, Cockermouth, Cumbria, UK) were connected to differential thermostats which maintained the surface temperature a constant 2–3 °C above ambient. Thus, the elevated temperature plots experienced the same variation in temperature, day length, light quality and humidity that the control plots experienced, the only difference being that the temperature was constantly elevated by 2–3 °C. Cables were placed in the surface of the plot, in between each row of plantings. Data loggers (Hobo U2 Temperature data loggers) were set up to record temperatures in both treatments. The plots were treated with Roundup™ herbicide (Scotts HQ, Marysville, OH, USA) 3 weeks prior to planting then tilled and levelled 1 week later.

Plants were inspected daily, and seedlings within a single planting ring were thinned down to a single one 10 days after planting. Any seed that germinated after this could not have been detected and thinned because the plant density prevented close inspection without damage to the growing plants. Flowering time was recorded as the first day an open flower was visible. At flowering time, we estimated the plant's vegetative size by measuring the rosette diameter. The diameter of each plant's rosette was measured across two perpendicular axes, and the estimated diameter was calculated as the average of the two measures. The same approximate axes were used for all plants, by orienting a ruler at a 45° and 135° angle from the plant label. After senescence, all plants were harvested and the number of fruits on each plant (larger than 1 cm) was counted in the laboratory to estimate fitness. When more than one seedling was present in a single planting ring, we phenotyped the largest plant because the smaller plants were more likely to have been the later germinants.

Because we were interested in the effect of temperature on phenology in a complex environment with multiple environmental cues, we analysed flowering time both in terms of number of days from planting to flowering, and in terms of ‘photothermal time’ or PTT, which was calculated as 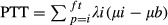
(Brachi *et al.*, 2010), where *p* is the planting date; *ft* is the flowering date; μ*i* is the mean daily temperature during daylight (calculated from the data collected from the data loggers); μb is the base temperature for development (estimated to be equal to 3 °C, Granier *et al.*, 2002); and λi is the daily photoperiod as a proportion of 24 h (photoperiod data were extracted from www.timeanddate.com). The PTTs represent the threshold number of photothermal units (PTUs) needed for a genotype to flower. This threshold is expected to be determined genetically, and independently of the environment, in which a genotype is grown, if the model is correct. Here, as the only difference between the two treatments is temperature, no difference in flowering time when measured in PTUs is expected across treatments if time to flower is a linear function of temperature, and temperature does not alter the threshold.

### Data analysis

All statistical analyses were performed in *R* version 2.13.0. We initially tested for significant effects of temperature on germination and survivability to flowering using logistic regression using all data available. As temperature had no significant effect on germination or survivability to flowering (Results), the subsequent analyses were performed on 278 lines for which we have a complete data set for three or more replicates. All replicates of these 278 lines that died after germinating without producing fruits were assigned a fitness of zero and included in the analysis.

To determine the effect of elevated temperatures on flowering time, rosette size, and fruit number; as well as genetic variation and genotype-by-environment interactions for these traits, we fitted the following mixed effects model using the *R* package *lme4*(Bates & Maechler, 2009): Trait = Treatment + MAGIC line +Treatment × MAGIC line + Density + Edge + Plot(Treatment) + error. In this model, treatment was set as a fixed effect, whereas genotype was set as a random effect. Plot was set as a random effect nested within treatment. Each replicate was scored as being on the edge of a plot or not, and edge effects were controlled for as a fixed effect. We controlled for density effects for each data point in all models by setting density (number of plants within a planting ring) as a fixed effect. MCMC p-values (10000 MCMC samples) were calculated for the fixed effects using *pvals.fnc* from the *R* package *languageR*(Baayen, 2008). Significance of random effect variance components were determined by likelihood ratio tests. To provide the final unbiased variance components, the model was then re-fitted using restricted maximum likelihood (REML).

To estimate whether genotypic trait values under ambient temperatures could predict responses in elevated temperatures, we calculated cross-environment genetic correlations as the Pearson correlation coefficients of the best linear unbiased predictors (BLUPs) for the MAGIC line random effects of each trait. Standard errors for genetic correlations were obtained by jackknife.

To determine whether phenological sensitivity (i.e. the magnitude and direction of the plastic response in flowering time to elevated temperature) was positively associated with fitness under elevated temperature, we estimated the average phenological response of each MAGIC line as its flowering plasticity (equal to the mean flowering time in the control, minus in the elevated temperature plots). We then regressed the mean fruit production under elevated temperature for each line against its flowering time under elevated temperature and its phenological response (as in Weinig *et al.*, 2006 and Springate *et al.*, 2011). The choice of regressing fruit production under the elevated temperature is because we are specifically interested in testing whether phenological responsiveness is adaptive under climate change. We also included the mean flowering value under elevated temperature in the model, to separate the effect of the plastic response from the trait mean value. To determine if any association was a general association with more plastic genotypes, or specific to the phenological response, we also performed the same analysis using plasticity in rosette diameter.

### QTL mapping

We used a quantitative trait loci approach to determine if the genetic architecture underlying flowering time and fitness was affected by elevated temperatures. We used the BLUPs for the MAGIC lines from a REML mixed effect model to give estimates of the line effects for each trait. The use of BLUPs was chosen due to the small effects detected for edges, plot and density (see results). The model used to generate the BLUPs was: Trait = MAGIC line + plot + Edge + density, with MAGIC line set as a random effect, and the others as fixed effects. The models were run for all traits in both ambient and elevated temperature treatments separately. To determine if there were QTL affecting the plastic response of traits independent of QTL that directly affected the trait value, we also mapped QTLs for the magnitude of the plastic response. QTL were mapped using the method described in Kover *et al*. (2009b), where a probabilistic reconstruction of the haplotype mosaic of each MAGIC line is initially calculated, and the genome is scanned for evidence of a QTL in each SNP interval using a fixed effects model. The genome-wide evidence in favour of a QTL was evaluated by resampling the data 500 times and fitting multiple QTL models.

## Results

In the first week of the experiment, daytime temperatures averaged 14.9 °C and night time temperatures averaged 10.7 °C. At the end of the experiment, 3 months later, the average temperatures were 16.2 °C and 14.8 °C respectively. Day length varied during the experiment from 14 to 17 h long. During the whole experiment elevated plots were on average 2.6 °C warmer.

Germination was high in both control and elevated plots (87.5% and 87.9% of the plantings respectively). Mortality was overall quite sporadic, with 96% of the plantings that have germinated surviving to flowering in the control plots and 98.5% in the elevated plots. There was no significant effect of temperature treatment on either germination (χ^2^ = 3.1, *P*_(*df* = 1)_ = 0.08), or survival of seedlings to flowering (χ^2^ = 1.0, *P*_(*df *= 1)_ = 0.31).

**Fig 1:**
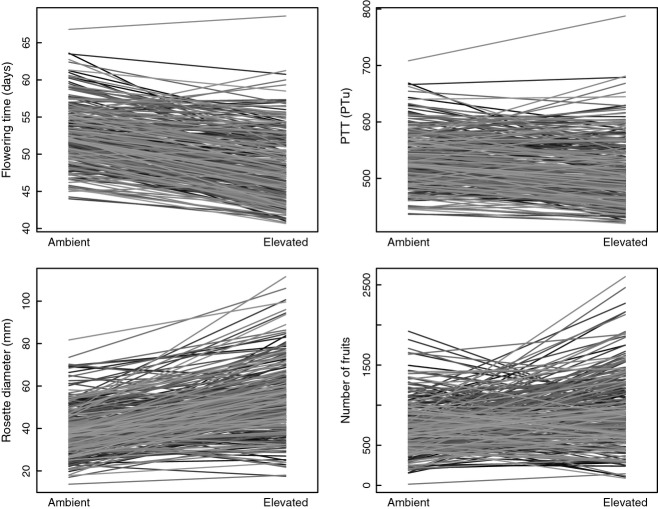
Mean reaction norms between ambient and elevated temperature treatments for four traits in *A. thaliana* MAGIC lines.

Elevated temperature significantly affected days to flowering time, rosette diameter and number of fruits: Plants under elevated temperature, on average, flowered ca. 4 days earlier, had rosettes ca. 13 mm larger and produced ca. 200 more fruits (Table1). However, there was no significant effect on flowering time in terms of photothermal units, suggesting that temperature effects on flowering time is consistent with photothermal models. The effect of plant density, plot and edge effects were significant (Table S1) but explained a small amount of variation (*R*^2^ < 5%). Significant genetic variance was observed for all traits as indicated by the between MAGIC line variance (*V*_*g*_) (Table2). No significant interaction between line and treatment was observed for rosette diameter (*P* = 0.254), suggesting that most lines respond similar to elevated temperatures. In contrast, a significant line by treatment interaction was observed for number of fruits (*P* = 0.019), and a marginally significant effect was detected for flowering time (days to flowering *P* = 0.071; PTT *P* = 0.072). Norms of reaction for all four traits is shown in Fig.1.

**Table 1:** Mean trait values (and SE in parenthesis) under ambient and elevated temperature treatments. Mean Squares (MS), *F* statistic (*F*) and probability (*P*) values are for the effect of the elevated temperature treatment. Results for all variables included in the model are shown in Table S1. *P* values were calculated using MCMC resampling (*df *= 1)

Trait	Control	Elevated	MS	*F*	*P*
Rosette diameter (mm)	36.69 (2.54)	49.92 (3.41)	4219.6	13.2	**0.004**
Flowering time					
Days	53.15 (0.77)	49.02 (1.04)	255.5	14.5	**0.003**
PTT	540.51 (9.45)	522.9 (12.69)	4067.8	1.5	0.205
Number of fruits	786.5 (52.81)	989.73 (68.78)	3053558.0	9.8	**0.023**

Bold values indicate significant at *P* < 0.05.

**Table 2:** Variance components [genetic (*V*_*g*_*,)* and genotype-by-environment variance (*V*_*gXe,*_)], heritabilities (*H*^*2*^) and cross-environment genetic correlations (*R*_*g*_) for MAGIC lines grown under ambient and elevated temperature treatments

			Ambient	Elevated	
Trait	*V*_*g*_	*V*_*gXe*_	*H*^*2*^ (SE)	*H*^*2*^ (SE)	*R*_*g*_ (SE)
Rosette diameter	84.7^*^^*^	6.21	**0.15 (0.03)**	**0.28 (0.04)**	0.45 (0.39)
Flowering time (days)	11.1^*^^*^	0.71	**0.45 (0.04)**	**0.37 (0.04)**	**0.64 (0.04)**
Flowering time (PTT)	1677.43^*^^*^	113.19	**0.46 (0.04)**	**0.35 (0.05)**	**0.64 (0.04)**
Number of fruits	30678.1^*^^*^	18703.1^*^	0.10 (0.4)	**0.16 (0.04)**	**0.23 (0.05)**

Significance levels of genetic variances (determined by likelihood ratio tests) are indicated by asterisks (^*^P < 0.05, ^*^^*^P < 0.0001). Components in bold have *P* < 0.05.

**Fig 2:**
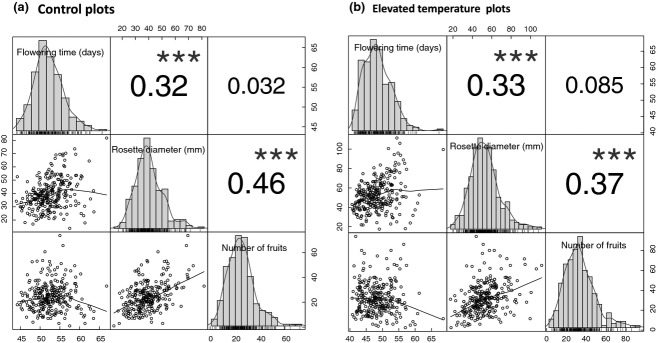
Correlations between mean trait values within treatment. Panel 2a shows values for the ambient treatment, and panel 2b for elevated temperature. Histograms and kernel density plots of the univariate distributions are shown on the diagonal. Pairwise Pearson correlations with starred significance levels are shown on the right of the diagonal. Scatter plots of the correlations with LOESS smoothers are shown left of the diagonal.

Pairwise genetic correlations among traits (based on genotype mean trait values) for ambient and elevated temperature treatments are shown in Fig.2 (PTT is not included because it is perfectly correlated with flowering time within treatments). Rosette diameter is significantly correlated with number of fruits in both treatments, meaning that larger plants also have more fruits. In contrast, flowering time is not significantly correlated with number of fruits, even though flowering time is positively correlated with rosette diameter – confirming that within treatment plants that flower earlier tend to have smaller vegetative size.

A significant correlation between fruit number under elevated temperatures and flowering plasticity shows that lines that are more phenologically responsive also tended to have higher fitness under elevated temperature (Table3). It is interesting to notice that flowering time under elevated temperatures did not have a significant direct effect on fruit production. In contrast, rosette diameter had a significant direct effect on fruit production under elevated temperature, but plasticity in rosette diameter did not (Table3).

**Table 3:** Regression of fruit number (fitness) under elevated temperature on mean trait value and its plasticity. Plasticity was measured as the difference between elevated and control means, and represents the response to changes in the temperature of the same genotype. Analyses were done twice using flowering time (measured in days) and rosette diameter

	Flowering (days)			Rosette diameter (mm)		
	*b*	*t*	*P*	*b*	*t*	*P*
Mean in elevated plots	−8.0 ± 6.8	1.2	0.237	10.1 ± 2.1	4.8	**3.0 × 10**^**−06**^
Plasticity	−40.9 ± 9.8	4.2	**3.9 × 10**^**−05**^	3.9 ± 2.4	1.6	0.102

Bold values indicate significant at *P* <0.05.

**Fig 3:**
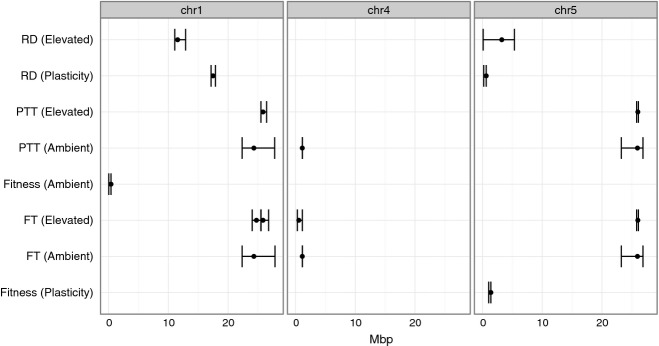
Positions of each QTL identified in this study. Only chromosomes 1, 4 and 5 (where QTL were identified) are shown. Further details about each of these QTL can be found on supplementary materials (Table S2).

Full genome LOD scans identified a total of 18 QTL for traits in ambient and elevated treatments, as well as for plasticity (Fig.3, Table S1 in the supporting material). Three QTL were detected for days to flower in ambient temperatures in chromosomes 1, 4 and 5. While under elevated temperatures 4 QTL were observed, the only difference is that in the elevated temperature treatment 2 distinct QTLs are detected on chromosome 1 (where a broader peak in the same location is observed in the controls). No QTL for plasticity in flowering time was detected. All QTLs for PTT overlapped with those for flowering time (Fig.3). Two QTL for rosette size were detected in the elevated temperature treatment on chromosomes 1 and 5. No QTLs for rosette size were detected in the control plants, but two QTLs for rosette size plasticity were detected on chromosomes 1 and 5. The QTL for rosette size plasticity on chromosome 1 did not colocate with a QTL for rosette diameter itself, suggesting that there is some separate genetic control for trait value and its plasticity in rosette size. One QTL was found for number of fruits on chromosome 1 under control conditions, but no colocated QTL for fruit number is seen under elevated temperatures (Fig.3). A QTL for fruit number plasticity is observed in chromosome 5, suggesting again separate genetic control from trait value.

## Discussion

We used a climate warming simulation approach and a set of RILs to investigate the effect of small increases in mean temperature on the phenology and fitness of *A. thaliana,* in a complex environmental background. We found that plants that experienced elevated temperatures flower significantly earlier than plants in control plots, in agreement with previous studies that compared flowering time across years or different locations (Fitter & Fitter, 2002; Menzel *et al.*, 2006; Anderson *et al.*, 2012). It is noteworthy that in this study, the changes in average temperature were quite small (<3 °C), and many other cues could be used to trigger flowering (such as daily variations in temperature, seasonal increase in day length, precipitation, etc.). Although plants flower significantly earlier, we found that, on average, plants flower at similar thresholds of PTUs, suggesting that photothermal models (e.g. Brachi *et al.*, 2010; Chew *et al.*, 2012) provide a good approximation of the effect of temperature on flowering under natural conditions. This is important because it indicates that the effect of temperature in climate change models can be efficiently modelled by a predictable linear relationship. Although the average change is well captured by the photothermal model, it is important to notice that some genotypes show clear deviation from the expected reduction in days to flowering time (Fig.1), and the test for GXE effects approaches significance (Table S1). Thus, it should be interesting in the future to pursue the genetic basis of the few lines that were identified to have uncharacteristic norms of reaction.

Plasticity is the result of differential phenotypic expression of the same genotype in response to different environments. Here, we detected significant differences in the flowering time, rosette size and fruit production of MAGIC lines under elevated temperature. Understanding the genetic basis of variation in plasticity could be useful in developing genetic models of plant response to climate change (Chevin *et al.*, 2010; Nicotra *et al.*, 2010). The different phenotypic expression may be the result of a different set of QTL underlying the trait under different environments (i.e. some genes are expressed only under some circumstances, or some gene function is only relevant under some environments) (Gardner & Latta, 2006; Lacaze *et al.*, 2008). Alternatively, it may be due to the existence of a ‘plasticity gene’ that affects the magnitude of the plastic response independent of the QTL that affect variation in trait value (Schlichting & Pigliucci, 1993). Here, we found that plasticity may have different genetic causes depending on the trait. A distinct QTL was observed for rosette size and fruit number plasticity that does not correspond to the QTL identified for trait mean value, suggesting the existence of genetic factors that affect the plasticity of a trait somewhat independently of genes that affect the trait value. In contrast, the same QTL for flowering time were identified under both treatments, whether flowering was measured in terms of days or in PTUs. These results suggest that a common genetic pathway regulates flowering time under ambient and elevated temperature, and that temperature affects flowering in a linear and predictable way. The plastic response in term of days to flowering appears to be a simple consequence of an accelerated accumulation of PTUs with higher temperature without significant changes in the genetically determined threshold.

There has been concern that accelerated phenology could reduce yield and fitness because an earlier trigger in the transition to flowering could constrain vegetative development, leading to smaller rosettes and lower seed production (Lobell & Field, 2007; Craufurd & Wheeler, 2009). However, although we observe a positive relationship between rosette size and flowering time within treatments (confirming life-history theory predictions), across treatments this pattern is not upheld. Plants growing under elevated temperature flower earlier but at a larger vegetative size than plants under ambient temperature, leading to an increase in fitness. This may be explained by faster development due to improvement of growing conditions with the increase in temperature during spring, so that flowering occurred earlier without compromising vegetative growth. A similar effect of higher temperatures on the rate of leaf production has been previously reported in laboratory experiments (Granier *et al.*, 2002; Hoffmann *et al.*, 2005). It is possible that the same effect would not have been observed if the experiment was run later in the summer or in a different geographical region where the increase in temperature would put averages above ideal temperatures for *A. thaliana* growth in late spring. Further experiments should investigate the effect of season and geographical location on the generality of our results.

Early flowering in *A. thaliana* has been proposed to be favoured in spring (Korves *et al.*, 2007), so the increase in fitness under elevated temperature could have simply resulted from earlier flowering. However, we found that flowering time was only weakly correlated with fitness in either treatment (*R* < 0.1). In contrast, we found that rosette diameter is more strongly correlated with number of fruits, and that elevated temperatures cause an increase in rosette size. Thus, the increase in fruit production (and therefore, fitness) due to elevated temperature is most likely mediated by the increase in vegetative size due to faster development.

Although on average there is an increase in fitness under elevated temperature, this response was not consistent across genotypes, as we detect a significant line by treatment interaction for fruit production. Most of this interaction is due to changes in the rank order of genotypes (Fig.1). The low genetic correlation across treatment in fitness means that the current distribution of fitness is a poor predictor of future fitness ranking in responses to climate warming. This conclusion is further supported by the fact the QTL for fitness identified in ambient temperatures is not observed under elevated temperatures. It follows that current populations would likely be maladapted if the environment change by an average of approximately 3 °C, and populations are unable to respond through genetic adaptation. For plant species with small generation times and large populations, such as *A. thaliana*and many other spring annuals, a slow increase in temperature is likely to result in significant changes in population genetic composition.

There is concern that mismatch in responses to climate change between plants and their pollinators may cause severe fitness decrease for some species (Hegland *et al.*, 2009). Because *A. thaliana* can self-fertilize, the observed fitness response to temperature in this study is unconstrained by the need to coordinate its flowering times with other mating plants or pollinators. In addition, the fact that our experiment was carried under a fruit cage, minimize any impact herbivores could have had. Thus, it is important to notice that in our experiment, any impact of a mismatch between *A. thaliana* and other members of the community on fitness was minimized, and the effect of elevated temperature on fitness might be overestimated. Although recent work suggest that the dangers of asynchrony might be compensated by a diverse community of pollinators and herbivores (the biodiversity insurance hypothesis, e.g. Bartomeus *et al.*, 2013), broad generalization of our results will require further studies using plants with other mating characteristics and different phenological schedule.

Identification of genetic mechanisms that help plants cope with environmental changes will improve the likelihood that populations would persist long enough for the necessary genetic adaptation to take place. Plastic responses might provide a buffer for the effect of climate change and help populations persist (Chevin *et al.*, 2010; Nicotra *et al.*, 2010). Here, we identified a QTL for plasticity in fruit number, where plants with an allele from the Ws-0 accession at this location produce ca. 500 more fruits under elevated temperatures, whereas plants with an allele from the Rsch-4 accession produces ca. 100 fewer fruits (Table S2). Although a previous study has observed environment-specific QTL for fruit production in the field (Weinig *et al.*, 2003), we are not aware of any previous studies that identified QTL for the magnitude of the plastic response in fitness. Further studies on the nature of this QTL in *A. thaliana* may provide significant insight in mechanisms that mediate fitness response to environmental changes. Investigation of currently annotated genes under the QTL peak identifies *DREB2* (At5G05410) as a possible candidate gene. This gene is known to respond to temperature and to affect growth (Yoshida *et al.*, 2011), but has not been previously associated with fitness under elevated temperatures.

In summary, the novel combination of temperature manipulation in the field with the use of MAGIC lines in this experiment has allowed us to determine that plasticity may have an important role in a plant's ability to cope with climate change. While on average plants under elevated temperature had higher fitness, not all genotypes responded equally, and some clearly declined in fitness. We found evidence for natural genetic variation for adaptive plasticity that significantly affects fitness ranking. Thus, significant changes in the genetic composition of populations are likely in response to imminent climate changes. The magnitude of this change, and whether populations will find themselves maladapted, will depend on the population size and the species’ generation time. Annual plants such as *A. thaliana* may be able to respond quickly given their large progeny and short generation time, but plants with longer generation or smaller populations may experience significant reduction in fitness. These results suggest that when plasticity is included in models of species persistence under climate change, it is important to also consider that there is genetic variation in plastic responses. Future work should further investigate the genetic mechanisms underlying plastic responses that increase fitness under climate change, since depending on pleiotropic effects of these genetic factors, the phenological and ecological characteristics of the populations can be significantly changed. Nevertheless our results do support the idea that genotypes that are more phenologically responsive tend to be more successful under elevated temperatures, suggesting that phenological sensitivity can be a useful indicator of genotypes to be used in restoration and conservation projects.

## Supporting Information

Table S1 *P* values (calculated using MCMC resampling) for all variables included in the general linear model run to determine the effect of elevated temperature (treatment), Genotype (Line), density, edge and plot.

Table S2 List of all QTL identified for traits measured, indicating chromosomal location (Chr), position in Kb and likelihood of QTL (log P). The estimated effect of having one of the 19 possible haplotypes at each of the QTLs is also listed under the columns with the parental accessions name. Accession number and more detail of each of these natural accessions available from Kover *et al*., 2009b. The largest allele effect, increasing the value of the trait, is underlined. The allele effect that causes the smallest trait value is highlighted in bold.
