# Supplementary material for: Plant responses to elevated temperatures: a field study on phenological sensitivity and fitness responses to simulated climate warming
Source: Glob Chang Biol. 2013 Nov 19;20(2):456–65. doi: 10.1111/gcb.12430 (PMC4253038; doi:10.1111/gcb.12430)

## Supporting material

1. Table S1: P values (calculated using MCMC resampling) for all variables included in the general linear model run to determine the effect of elevated temperature (treatment), Genotype (Line), density, edge and plot.

| Trait | treatment | Line | Treat X line | Density | edge | plot |
| --- | --- | --- | --- | --- | --- | --- |
|  |  |  |  |  |  |  |
| Flowering (days) | 0.003 | <0.001 | 0.077 | 0.0001 | 0.005 | <0.001 |
| Flowering (PTUs) | 0.205 | <0.001 | 0.073 | 0.0001 | 0.004 | <0.001 |
| Rosette diameter (mm) | 0.004 | <0.001 | 0.250 | 0.0001 | 0.139 | <0.001 |
| Number of fruits | 0.023 | 0.0003 | 0.019 | 0.0010 | 0.565 | <0.001 |

## Table S2: List of all QTL identified for traits measured, indicating chromosomal location (Chr), position in Kb, and likelihood of QTL (logP). The estimated effect of having one of the 19 possible haplotypes at each of the QTLs is also listed under the columns with the parental accessions name. Accession number and more detail of each of these natural accessions available from Kover et al 2009. The largest allele effect increasing the value of the trait is underlined. The allele effect that causes the smallest trait value in highlighted in bold.

| Treatment | chr | Pos  (Kb) | logP | Bur | Can | Col | Ct | Edi | Hi | Kn | Ler | Mt | No | Oy | Po | Rsch | Sf | Tsu | Wil | Ws | Wu | Zu |
| --- | --- | --- | --- | --- | --- | --- | --- | --- | --- | --- | --- | --- | --- | --- | --- | --- | --- | --- | --- | --- | --- | --- |
| Rosette diameter | | |  |  |  |  |  |  |  |  |  |  |  |  |  |  |  |  |  |  |  |  |
| Elevated | 1 | 11554 | 4.26 | 5.0 | 4.7 | 2.0 | -1.4 | -0.7 | **-4.7** | -0.6 | -2.2 | -0.7 | 2.4 | -2.7 | 1.5 | 5.5 | -4.3 | 4.1 | -1.4 | -1.7 | 2.8 | 1.7 |
| Elevated | 5 | 3228 | 5.19 | -0.2 | 5.7 | 2.9 | -1.2 | 12.5 | -3.8 | -1.5 | -4.1 | 1.1 | -1.7 | 2.0 | 1.3 | 2.2 | 6.9 | 1.6 | -0.4 | 3.2 | **-5.1** | 3.4 |
| Plasticity | 1 | 17474 | 3.58 | 13.8 | 16.8 | 10.6 | 12.0 | 11.6 | **6.9** | 14.4 | 11.5 | 15.0 | 14.2 | 10.9 | 15.4 | 19.9 | 10.5 | 22.8 | 7.7 | 17.8 | 16.7 | 12.8 |
| Plasticity | 5 | 626 | 3.62 | 14.8 | 12.3 | 16.4 | 21.5 | 21.2 | 10.1 | 12.6 | 5.2 | 20.6 | 13.5 | 19.1 | 12.8 | 16.4 | 17.2 | 15.1 | 9.1 | 11.9 | **4.9** | 14.5 |
| Flowering time (days) | | |  |  |  |  |  |  |  |  |  |  |  |  |  |  |  |  |  |  |  |  |
| Ambient | 1 | 24319 | 6.85 | -0.6 | -0.4 | -0.8 | 1.7 | 2.7 | -1.1 | **-1.4** | -0.1 | -1.1 | 0.3 | 0.4 | -0.3 | -0.8 | -0.6 | 2.9 | -0.6 | 0.9 | -0.6 | -0.4 |
| Ambient | 4 | 1124 | 3.83 | 0.9 | 3.4 | -1.1 | 0.1 | 1.4 | -0.6 | 1.0 | -0.7 | 0.9 | 0.2 | -0.6 | 0.5 | -0.4 | 2.5 | 0.9 | **-1.3** | -0.6 | 0.8 | -0.4 |
| Ambient | 5 | 25947 | 6.61 | 0.1 | -1.5 | -0.7 | -1.1 | 0.1 | -0.9 | 0.2 | 0.8 | -0.2 | **-1.6** | 1.6 | 1.7 | 0.9 | -0.7 | 0.6 | -1.0 | -1.3 | 2.2 | -0.2 |
| Elevated | 1 | 24749 | 3.78 | -0.6 | -0.7 | 0.0 | 1.7 | 1.3 | -1.4 | -0.9 | -0.5 | -0.7 | 0.3 | 1.0 | 1.3 | -0.1 | -0.8 | 2.0 | -1.2 | 1.2 | **-1.5** | 0.6 |
| Elevated | 1 | 25850 | 4.45 | -0.4 | -0.3 | 0.0 | 1.4 | 0.5 | **-1.5** | -1.2 | -0.5 | -0.9 | 0.7 | 0.6 | 1.1 | 0.3 | -0.5 | 1.8 | -1.5 | 0.8 | -1.3 | 1.1 |
| Elevated | 4 | 541 | 3.63 | 0.6 | 3.0 | -0.9 | -0.2 | 1.5 | -0.6 | 1.7 | **-1.1** | 0.1 | -0.9 | 0.0 | 0.1 | -0.4 | 2.3 | -0.4 | -0.2 | 0.0 | 0.7 | -0.5 |
| Elevated | 5 | 26012 | 3.85 | -0.3 | -1.0 | -1.1 | -0.3 | -0.1 | -1.5 | 0.5 | 0.4 | -0.6 | **-1.8** | 1.1 | 1.1 | 0.6 | -0.1 | 0.7 | -0.2 | -1.1 | 2.4 | 0.3 |
| Photothermal time (PTT) | | |  |  |  |  |  |  |  |  |  |  |  |  |  |  |  |  |  |  |  |  |
| Ambient | 1 | 24319 | 6.82 | -7.2 | -5.1 | -9.6 | 21.6 | 33.2 | -13.9 | **-16.8** | -2.2 | -13.9 | 3.2 | 4.8 | -5.3 | -9.7 | -6.2 | 36.1 | -7.4 | 11.0 | -7.6 | -5.4 |
| Ambient | 4 | 1124 | 3.78 | 11.7 | 42.9 | -13.6 | 0.8 | 16.4 | -8.0 | 13.1 | -8.2 | 11.5 | 1.4 | -7.7 | 7.8 | -4.4 | 31.2 | 11.6 | **-15.9** | -8.3 | 10.2 | -4.1 |
| Ambient | 5 | 25946 | 6.74 | 1.1 | -18.0 | -8.6 | -12.8 | 0.7 | -11.9 | 1.8 | 10.2 | -3.5 | **-19.8** | 19.8 | 20.4 | 10.5 | -9.5 | 8.4 | -13.2 | -15.7 | 27.7 | -1.6 |
| Elevated | 1 | 25850 | 4.33 | -3.8 | -3.6 | -0.3 | 16.8 | 5.7 | **-18.1** | -13.4 | -6.2 | -10.4 | 7.8 | 7.0 | 13.2 | 3.5 | -5.8 | 22.0 | -17.1 | 8.7 | -16.1 | 12.2 |
| Elevated | 5 | 26012 | 3.82 | 12.0 | 47.9 | **-13.1** | -3.1 | 22.5 | -7.5 | 23.4 | -9.9 | 0.3 | -10.1 | -0.7 | -1.3 | -3.5 | 28.5 | -4.5 | -5.5 | -0.6 | 8.6 | -6.0 |
| Fruit Number (Fitness) | | |  |  |  |  |  |  |  |  |  |  |  |  |  |  |  |  |  |  |  |  |
| Ambient | 1 | 395 | 3.67 | -30.5 | 46.2 | 0.2 | -15.4 | -29.5 | 39.9 | -10.2 | 4.5 | 6.8 | -17.4 | 26.7 | 25.2 | -12.1 | 4.2 | 82.1 | -0.1 | **-46.0** | -28.0 | -30.3 |
| Plasticity | 5 | 1400 | 3.95 | 294.1 | 214.3 | 330.1 | 314.6 | 208.8 | 223.1 | 175.9 | 66.2 | 243.0 | -52.9 | 522.3 | 365.7 | **-131.7** | 367.9 | 29.4 | 53.0 | 509.2 | 16.4 | 421.4 |

Figure S1: Full genome scans for QTLs for flowering time traits in control and elevated temperature treatments


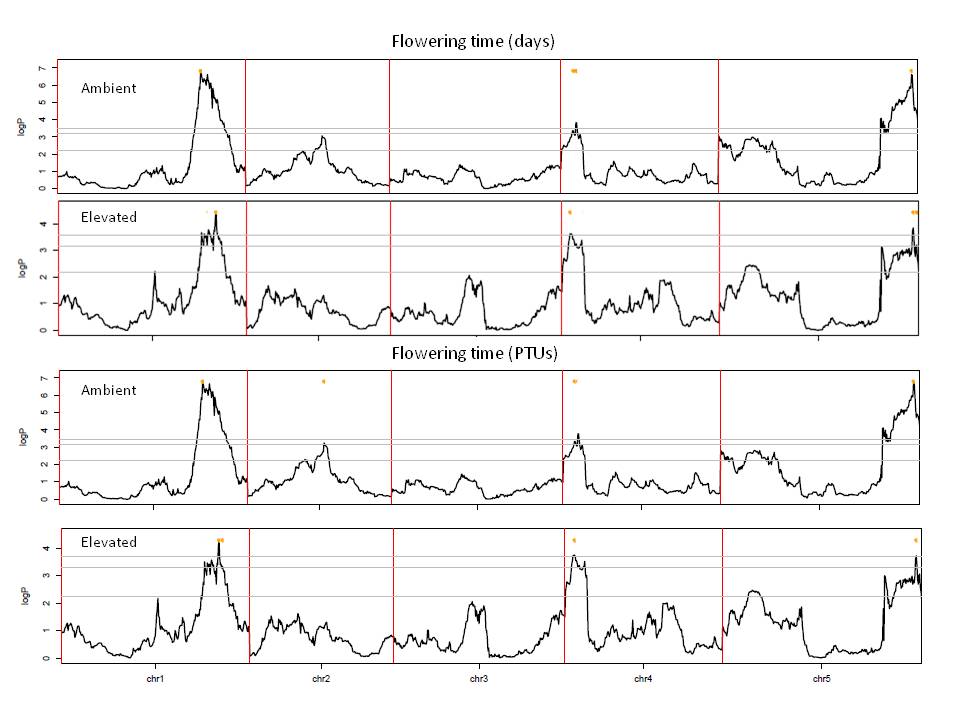


Figure S2: Full genome scans for QTLs for rosette diameter in ambient (A) and elevated (B) temperature treatments. Panel C shows genome scan for QTL in plasticity in rosette diameter.


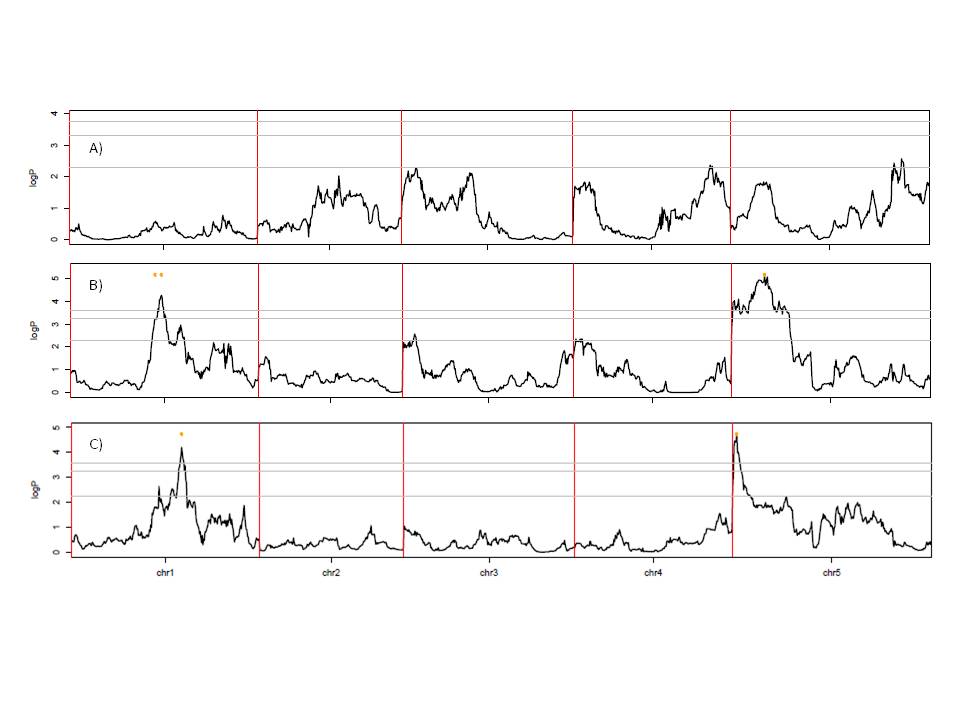


Figure S3: Full genome scans for QTLs for Fruit production (fitness) in ambient (A) and elevated temperature (B) treatments. Panel C shows genome scan for QTL in plasticity for fitness.


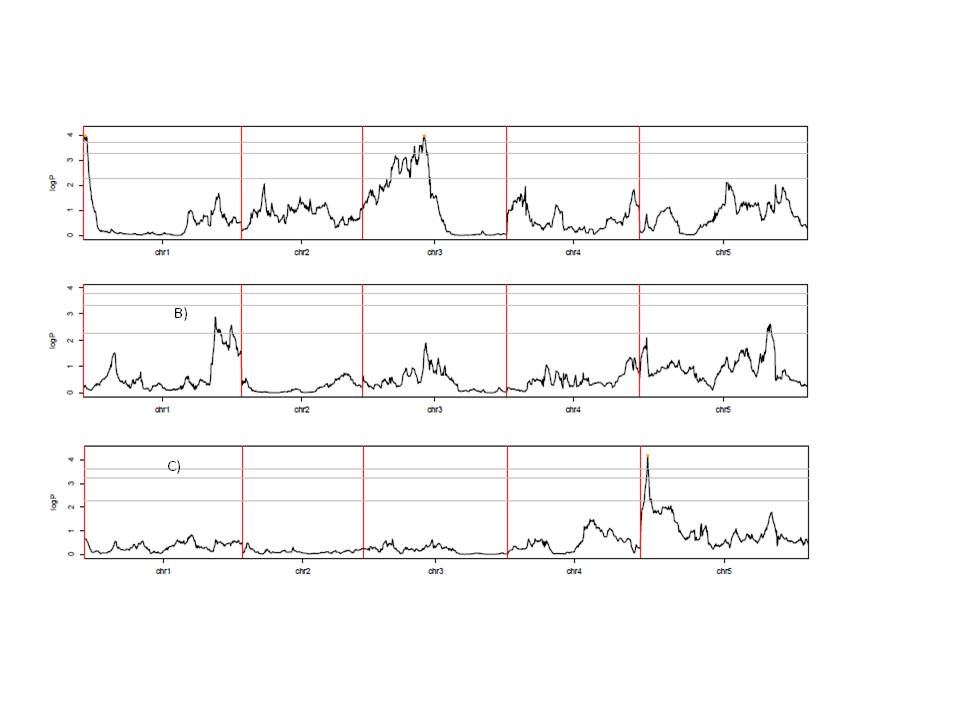

Supplement: Table S1 — P values (calculated using MCMC resampling) for all variables included in the general linear model run to determine the effect of elevated temperature (treatment), Genotype (Line), density, edge and plot. [file gcb0020-0456-SD1.docx]
